# Lactation in an Infant

**Published:** 1852-01

**Authors:** 


### LACTATION IN AN INFANT.

Dr. John Hastings, of San Francisco, California, has sent us the following brief notes of a case of lactation in an infant.

"June 22d, 1851.—Mrs. Quiefelt was delivered of a healthy female child weighing ten pounds, whose breasts gave milk freely from the day of its birth until the 9th of August following. The mother is a Swede, about twenty-seven years of age, and stout and healthy, this being her sixth child. Neither the mammary glands, nor the nipples of the child, were unusually developed."

California equals the "promised land" in the "overflowing of milk and honey."

